# Linear Tuning of Phase-Matching Temperature in LiNbO_3_:Zr Crystals by MgO Co-Doping

**DOI:** 10.3390/ma12244155

**Published:** 2019-12-11

**Authors:** Tengfei Kong, Hongde Liu, Liyun Xue, Weiwei Wang, Shahzad Saeed, Dahuai Zheng, Shiguo Liu, Shaolin Chen, Ling Zhang, Yongfa Kong, Jingjun Xu

**Affiliations:** 1 School of Sciences, Henan University of Technology, Zhengzhou 450001, China; ktfsunshine@mail.nankai.edu.cn; 2 MOE Key Laboratory of Weak-Light Nonlinear Photonics, School of Physics and TEDA Institute of Applied Physics, Nankai University, Tianjin 300071, China; 1120150044@mail.nankai.edu.cn (L.X.); weiweiwang@mail.nankai.edu.cn (W.W.); shehzadsaeed2003@yahoo.com (S.S.); dhzheng@nankai.edu.cn (D.Z.); nkliusg@nankai.edu.cn (S.L.); chenshaolin@nankai.edu.cn (S.C.); zhangl63@nankai.edu.cn (L.Z.); jjxu@nankai.edu.cn (J.X.)

**Keywords:** lithium niobate, phase-matching temperature, linear tuning, defect structure

## Abstract

We grew a series of co-doped LiNbO_3_ crystals with fixed 1.5 mol % ZrO_2_ and various MgO concentrations (1.0, 3.0, 4.0, 6.0 mol %), and investigated their optical properties and defect structures. By 3.0 mol % MgO co-doping, the optical damage resistance at 532 nm reached 6.5 × 10^6^ W/cm^2^, while the phase-matching temperature for doubling 1064 nm was only 29.3 °C—close to room temperature—which was conducive to realizing the 90° phase matching at room temperature by slightly modulating the incident angle of the fundamental beam. Notably, we found that the phase-matching temperature increased linearly with the increase of MgO doping, and this linear dependence helped us to grow the high-quality crystal for room temperature 90° phase matching. Moreover, the defect analysis indicated that the linear tuning of phase-matching temperature might be attributed to ${\mathrm{Mg}}_{\mathrm{Li}}^{+}$ + ${\mathrm{Zr}}_{\mathrm{Nb}}^{-}$ neutral pairs in crystals.

## 1. Introduction

Lithium niobate (LiNbO_3_, LN) is a well-known nonlinear optical material for frequency conversion applications, due to its large nonlinearity and capacity for noncritical phase matching in a variety of interactions in the visible and near-infrared [1,2]. However, the practical use of LN (e.g., as a harmonic generator) is severely limited by optical damage at high light intensities [3]. Recently, some studies have shown that LN co-doping with some impurities, such as Mg^2+^, Zn^2+^, In^3+^, and Zr^4+^, can substantially reduce the optical damage in the visible region above a certain threshold value [4,5,6,7,8]. Among these optical-damage-resistant additives, Mg^2+^ is the most commonly used dopant in LN at this time, and MgO-doped LN (MgLN) heavily promotes the practical applications in high-power frequency conversion [9,10,11]. However, MgLN must be heated to a temperature of ~110 °C to achieve efficient phase matching with the help of a temperature-controlled oven, unavoidably increasing the additional energy losses and the components’ instability [12,13].

Recently, we reported that the 1.7 mol % ZrO_2_ and 5.0 mol % MgO co-doped LN crystal not only realizes efficient room temperature 90° phase matching for doubling 1064 nm, but also exhibits high optical damage resistance at 532 nm [14]. Here, there are two reasons why we select Zr^4+^ and Mg^2+^ ions as double-doping candidates in LN. First, Mg^2+^ is widely used as a primary additive co-doping with other dopants in LN to precisely regulate optical properties and defect structures, such as LN:Mg,Bi [15] and LN:Mg,Zn [16]. These reports provided us with ideas to lower the phase-matching temperature while maintaining the optical damage resistance in LN. Second, Zr^4+^ ions in LN have a low doping threshold level and a distribution coefficient close to one; meanwhile, ZrO_2_-doped LN has excellent optical damage resistance properties, from ultraviolet to visible range [17,18]. However, it must be pointed out that the total doping concentration of ZrO_2_ and MgO in the melt for our double-doped LN is so high that it is bad for the growth of the high-quality crystal. Moreover, the specific roles of Zr^4+^ and Mg^2+^ in this co-doped crystal are still not clear. Therefore, exploring the more preferable doping concentration of ZrO_2_ and MgO in LN, to achieve the room temperature 90° phase matching for doubling 1064 nm, is worthy of further investigation. Furthermore, it is highly necessary to clearly understand the substitution process of Zr^4+^ and Mg^2+^ ions in LN for purposefully regulating the crystal properties.

In this work, we grew a series of LiNbO_3_ crystals by the conventional Czochralski method, co-doped with a fixed 1.5 mol % ZrO_2_ and various MgO concentrations, and investigated their optical damage resistance and phase-matching temperatures. At the same time, the UV-visible absorption spectra and the OH^−^ absorption spectra were employed to explore the defective structures of crystals.

## 2. Materials and Methods

All of the investigated crystals were pulled from a congruent melt to which ZrO_2_ and MgO were added by the conventional Czochralski method. The congruent composition Li/Nb was selected as 48.38/51.62. The concentration of ZrO_2_ was fixed as 1.5 mol % while the MgO concentrations were 1.0, 3.0, 4.0, and 6.0 mol %, respectively, labeled as LN:Zr,Mg_1.0_, LN:Zr,Mg_3.0_, LN:Zr,Mg_4.0_, and LN:Zr,Mg_6.0_. The as-grown crystals in the furnace at 1200 °C were annealed for 10 h and polarized under the density of 5 mA/cm^2^ for 1 h. After annealing treatment and polarization, crystal sheets that were 1.0 and 3.0 mm thick along the *Y* faces were prepared for the characterization of absorption spectra and optical damage resistance, respectively. The uniform and transparent crystals were also cut into cubes of 10 × 10 × 10 mm^3^, perpendicular to the crystal *Y*-axis for 90° phase matching. The direction of the *C*-axis was determined by the pyroelectric effect. For comparison, normally congruent pure and 2.0 mol % ZrO_2_-doped LiNbO_3_ crystals were grown under the same conditions, labeled as CLN and LN:Zr_2.0_, respectively. All samples were polished to optical grade. Here, no inclusions in the polished sheets were visible to the naked eye, and yet the slight growth stripes still existed as examined by a vertically polarized 671 nm laser.

The laser beam distortion experiment was employed to test the optical damage resistance. An *e*-polarized 532 nm laser irradiated the 3.0 mm thick *Y*-plates for 5 min. The laser beam was polarized parallel to the *C*-axis of the plates. Optical damage was induced by a transmitted light beam becoming smeared and elongated along the *C*-axis and by a decrease of the intensity of its central part. Then we also used the two-wave coupling method to measure the change of refractive index of the 3.0 mm crystal sheets, to quantitatively characterize the optical damage resistance. Two *e*-polarized coherent beams at 532 nm with equal intensities 400 mW/cm^2^ were intersected in the samples, with a crossing angle of *2θ_cry_*, 30° in the air. The grating vector was aligned along the *C*-axis of the samples to utilize the largest electro-optic coefficient *r_33_*. The diffraction efficiency *η* of the phase grating was given by the equation $\eta ={sin}^{2}(\pi d\Delta n/\lambda cos\theta_{cry})$ [19], where *η* is the diffraction efficiency, λ is the laser wavelength, *d* is the thickness of the crystal, and $\theta_{cry}$ is the intersecting half-angle of the two coherent beams outside the crystal. The change of the refractive index Δ*n* could be calculated from the aforementioned equation by experimentally measuring the diffraction efficiency.

The temperature for 90° phase-matched second-harmonic generation (SHG) was measured by using a 1064 nm Q-switched Nd:YAG pulse laser (Guangan laser, Hefei, China) with 1 Hz repetition rate and 8 ns pulse width. The laser facula diameter was 5.0 mm, and the pulse energy was 320 mJ. The fundamental frequency light was aligned with the crystal’s Y-axis, while its polarization direction was perpendicular to the crystal’s *C*-axis. The second-harmonic energy was detected by the pulse laser energy meter. The bulk sample was mounted in an oven thermally controlled to within ±0.2 °C. The distance between the entrance window and the exit window of the oven was at least 5.0 cm to minimize the possible temperature gradients along the crystal *Y*-axis. The temperature was measured with a Pt-100 thermistor placed in direct contact with the crystal. The equation for the SHG efficiency was *η* = *E*_2_/*E*_1_, with *E*_1_ and *E*_2_ as the fundamental energy and the second-harmonic energy, respectively.

In addition, to explore the micro-mechanism between the defective structures and the optical properties of the crystals, the UV-visible absorption spectra and OH^−^ absorption spectra were measured using a U-4100 spectrophotometer (Hitachi, Tokyo, Japan) and a Magna-560 Fourier transform infrared spectrophotometer (Nicolet, MMAS) respectively, with incident light transmitting 1.0 mm thick plates along the *Y*-axis at room temperature. The step-length of the UV-visible spectrometer was 1.0 nm, and the resolution of the infrared spectrophotometer was 4.0 cm^−1^.

## 3. Results and Discussion

### 3.1. Optical Damage Resistance

**Figure 1:**
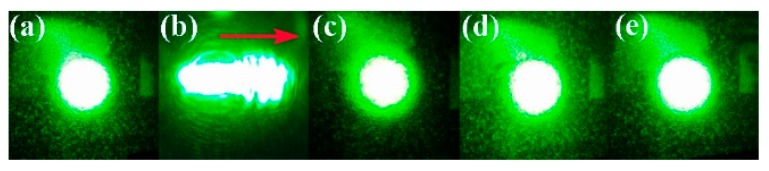
Distortion of transmitted laser beam spots after 5 min irradiation. The red arrow represents the *C*-axis of the crystal. (**a**) LN:Zr_2.0_; (**b**) LN:Zr,Mg_1.0_; (**c**) LN:Zr,Mg_3.0_; (**d**) LN:Zr,Mg_4.0_; (**e**) LN:Zr,Mg_6.0_. The light intensities are (**a**,**c**–**e**) 6.5 × 10^6^ W/cm^2^ and (**b**) 5.9 × 10^3^ W/cm^2^.

Figure 1 shows the transmitted beam spots for the different samples after 5 min irradiation. For LN:Zr,Mg_1.0_ (Figure 1b), the transmitted light spot obviously diffuses along the *C*-axis of the crystal under an intensity of 5.9 × 10^3^ W/cm^2^. As the MgO doping concentration increases to 6.0 mol %, the beam distortions of LN:Zr,Mg_3.0_ (Figure 1c), LN:Zr,Mg_4.0_ (Figure 1d), and LN:Zr,Mg_6.0_ (Figure 1e) decrease significantly, and these crystals can withstand the highest intensity of 6.5 × 10^6^ W/cm^2^ in our lab without noticeable beam smearing. Moreover, the conventional high optical damage resistant LN:Zr_2.0_ (Figure 1a) under the same conditions can also withstand a light intensity of 6.5 × 10^6^ W/cm^2^ without spot distortion.

**Figure 2:**
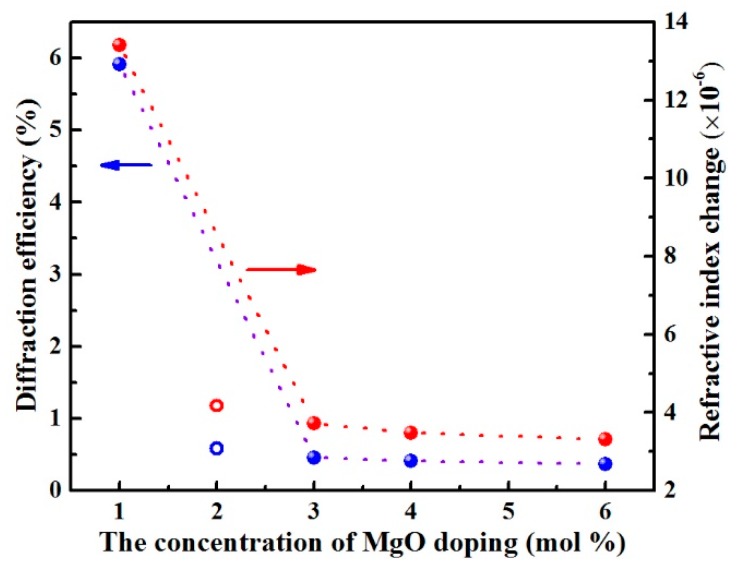
Dependence of the diffraction efficiency *η* and refractive index change Δ*n* of LN:Zr,Mg on the MgO doping concentration. For comparison, the open symbols represent the data of LN:Zr_2.0_. The dash lines are intended to guide the eyes.

The dependence of saturated diffraction efficiency *η* on the MgO doping concentration in LN:Zr,Mg is depicted in Figure 2. We can see that the saturated diffraction efficiency decreases with increased MgO doping concentration. LN:Zr,Mg_1.0_ has a saturated diffraction efficiency of 5.91%, and it abruptly decreases to 0.22% in LN:Zr,Mg_6.0_. Figure 2 clearly shows the relationship between the refractive index change Δ*n* and the MgO doping concentration. For comparison, under the same conditions the calculated saturated refractive index change Δ*n* of LN:Zr_2.0_ is 4.17 × 10^−6^. It can be seen from the figure that the refractive index Δ*n* of LN:Zr,Mg_3.0_, LN:Zr,Mg_4.0_, and LN:Zr,Mg_6.0_ are distinctly less than that of LN:Zr_2.0_, implying that the optical damage resistance of the former is even higher. We can derive from these data that MgO doping further reduces the refractive index change of crystals, while its effect is similar to that of the single optical damage resistant dopant. In addition, note that the 3.0 mol % MgO and 1.5 mol % ZrO_2_ co-doped LiNbO_3_ has almost the same optical damage resistance as the previously reported 5.0 mol % MgO and 1.7 mol % ZrO_2_ co-doped LiNbO_3_ [14].

### 3.2. The Temperature for 90° Phase Matching

**Figure 3:**
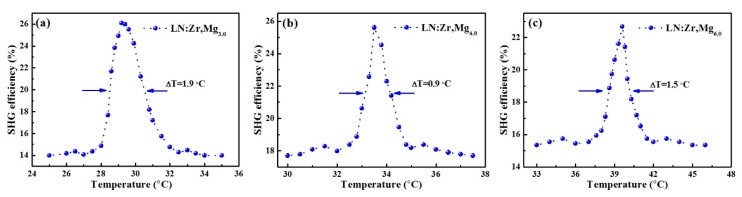
The second-harmonic generation (SHG) efficiency vs. the temperature for (**a**) LN:Zr,Mg_3.0_, (**b**) LN:Zr,Mg_4.0_, (**c**) LN:Zr,Mg_6.0_. The full width at half maximum (FWHM) of the temperature-tuning curve is also marked.

**Figure 4:**
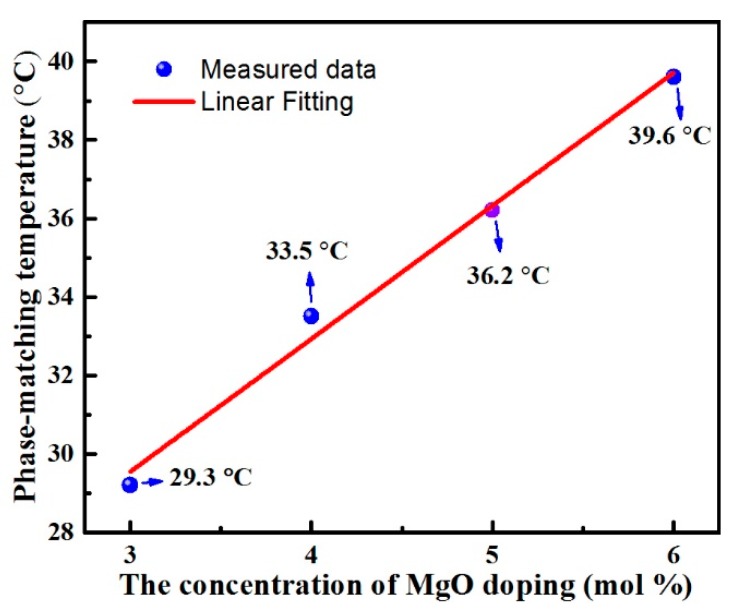
The phase-matching temperature as a function of MgO doping concentration for LN:Zr,Mg. The red line represents the linear fitting. The phase-matching temperatures of LN:Zr,Mg_3.0_, LN:Zr,Mg_4.0_, and LN:Zr,Mg_6.0_ are marked, respectively. The phase-matching temperature of LN:Zr,Mg_5.0_ [14] is also shown.

Temperature-tuned 90° phase matching for LN:Zr,Mg_3.0_, LN:Zr,Mg_4.0_, and LN:Zr,Mg_6.0_ was achieved by using a 1064 nm Q-switched Nd:YAG laser. Figure 3 illustrates the relationship between second-harmonic generation efficiency and the temperature, while the full width at half maximum (FWHM) of the temperature-tuning curve is also shown. From the figure, we know that the investigated crystals can achieve an efficient second-harmonic generation (SHG), and the SHG efficiency is maximized at the temperature for phase matching. Figure 4 describes the phase-matching temperature of LN:Zr,Mg as a function of the mole fraction of MgO doping in the melt. Moreover, to clearly elucidate the dependence between the phase-matching temperature and the MgO doping concentration, we also added the measured phase-matching temperature for the 1.5 mol % ZrO_2_ and 5.0 mol % MgO co-doped LiNbO_3_ crystal (labeled as LN:Zr,Mg_5.0_), as reported in our published paper [14]. As shown in the figure, the phase-matching temperature increases with increased MgO doping concentration, and the phase-matching temperature follows a linear function of the mole fraction of MgO doping in the melt. Therefore, based on this linear dependence we can adjust the phase-matching temperature of LN:Zr,Mg over a considerable range of temperature by the selection of the MgO doping concentration.

As we know, lower phase-matching temperature and higher optical damage resistance are essential conditions for developing simple, compact, and convenient visible lasers. Please note that the phase-matching temperature for LN:Zr,Mg_3.0_ is as low as 29.3 °C, close to room temperature (~25 °C), while the FWHM of the temperature-tuning curve is 1.9 °C. Here, it should be pointed out that the FWHM difference between the three test crystals may be caused by the quality of crystals. Moreover, the measured maximum SHG efficiency of LN:Zr,Mg_3.0_ is only 26.2%, and its low efficiency for doubling 1064 nm may be caused by the lack of anti-reflection coatings for the crystals’ Y-surfaces. These characteristics help to reduce the requirement of temperature control, which makes the phase matching at room temperature noncritical by slightly modulating the incident angle of the fundamental beam. In addition, we need to emphasize that the photochromic damage, called dark trace damage [20], is not observed when the crystals are exposed to 320 mJ energy of the Nd:YAG pulse laser for two hours. According to the aforementioned points, we can conclude that the LN:Zr,Mg_3.0_ crystal can be an excellent candidate for frequency conversion.

### 3.3. OH^−^ Absorption Spectra

**Figure 5:**
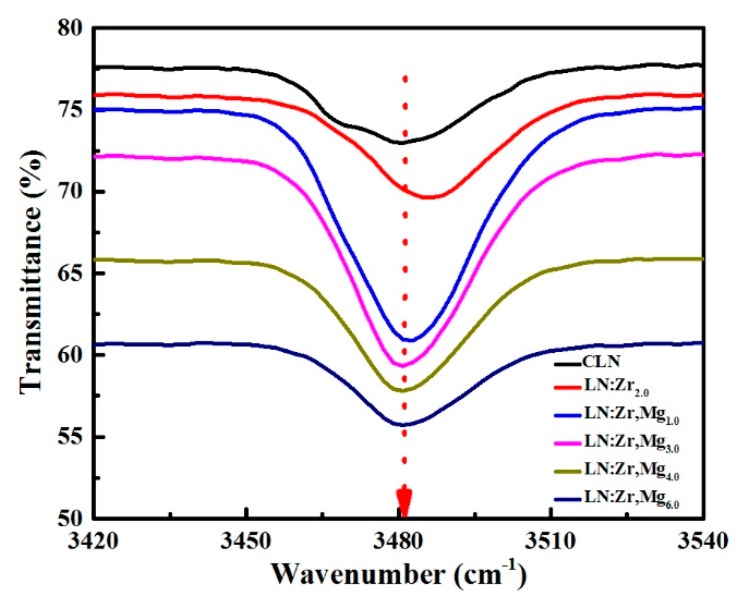
The OH^−^ absorption spectra of CLN, LN:Zr_2.0_, LN:Zr,Mg_1.0_, LN:Zr,Mg_3.0_, LN:Zr,Mg_4.0_ and LN:Zr,Mg_6.0_.

Figure 5 shows the OH^−^ absorption spectra of CLN, LN:Zr_2.0_ and LN:Zr,Mg crystals. It can be seen in the figure that the positions of the OH^−^ absorption bands of all samples are situated around 3484 cm^−1^. Due to the lack of a noticeable shift in the absorption bands, a three-peak fitting model [21] was adopted to precisely analyze the absorption spectra. The fitting results are listed in Table 1.

As shown in the table, the three peaks of CLN are situated at 3468 cm^−1^, 3481 cm^−1^, and 3490 cm^−1^, respectively, consistent with the previous literature [21]. In particular, the vibration 3468 cm^−1^ peak of LN:Zr,Mg_1.0_ moves to 3470 cm^−1^, and others move to 3473 cm^−1^, 3474 cm^−1^, and 3475 cm^−1^ for LN:Zr,Mg_3.0_, LN:Zr,Mg_4.0_, and LN:Zr,Mg_6.0_, respectively. The fitting peak of LN:Zr_2.0_ shifts from the position at 3468 cm^−1^ of CLN to 3475 cm^−1^. Generally, the absorption peak moving to 3473 cm^−1^ and the higher wavenumbers for ZrO_2_-doped LN can be considered as a signal of the doping threshold level, which is related to the formation of ${\mathrm{Zr}}_{\mathrm{Nb}}^{-}$ − OH^−^ complexes [22,23]. Thus, we infer that Zr^4+^ ions enter Nb sites to form ${\mathrm{Zr}}_{\mathrm{Nb}}^{-}$ defects in the LN:Zr,Mg_3.0_, LN:Zr,Mg_4.0_, and LN:Zr,Mg_6.0_. It is known that for MgO-doped LN the absorption peak at 3535 cm^-1^ is attributed to the vibration of ${\mathrm{Mg}}_{\mathrm{Nb}}^{3-}$ − OH^−^ complexes [24]. According to the measured absorption spectra, however, no 3535 cm^−1^ is observed in the investigated MgO and ZrO_2_ co-doped LiNbO_3_, which reflects the observation that Mg^2+^ ions only occupy Li sites in crystals. In addition, it is widely accepted that in doped LN a new absorption band related to the stretching vibration of $M_{\mathrm{Nb}}^{n+}$ − OH^−^ complexes appears when the doping concentration exceeds the threshold value, where $M_{\mathrm{Nb}}^{n+}$ is a di-, tri- or tetravalent optical-damage-resistant ion occupying an Nb site in the lattice [23,25]. Taken together, we can draw the conclusion that the total concentration of 1.5 mol % ZrO_2_ and 3.0 mol % MgO in LN has reached the doping threshold value. Meanwhile, by this analysis, we can also deduce that ${\mathrm{Mg}}_{\mathrm{Li}}^{+}$ + ${\mathrm{Zr}}_{\mathrm{Nb}}^{-}$ defect pairs without charge compensation by OH^−^ exist in crystals with the increase of MgO doping concentration from 3.0 mol % to 6.0 mol %, which is the reason that no absorption band obviously shifts.

### 3.4. UV-Visible Absorption Spectra

**Figure 6:**
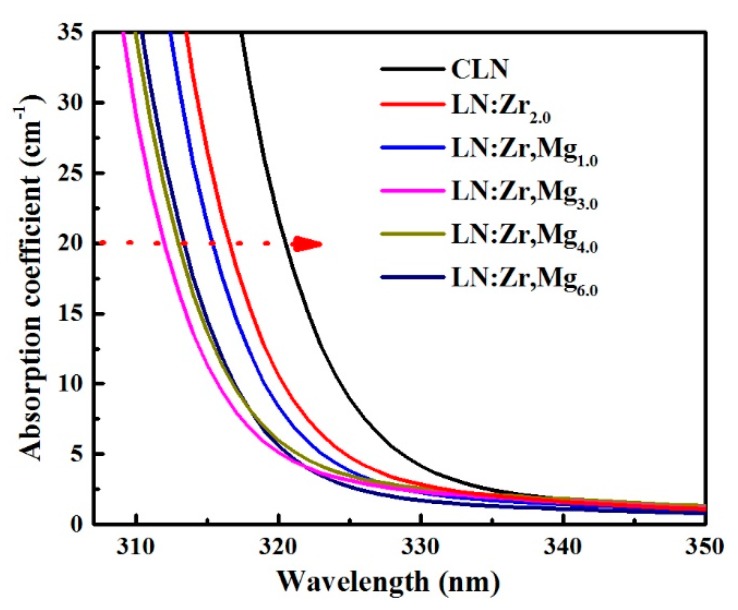
The absorption edges of LN:Zr,Mg_1.0_, LN:Zr,Mg_3.0_, LN:Zr,Mg_4.0_, and LN:Zr,Mg_6.0_; those of CLN and LN:Zr_2.0_ are also shown for comparison.

The absorption edge of LN is determined by the transition energy of the electron transition from O^2−^ 2*p*-state to Nb^5+^ 4*d*-state [26]. Figure 6 depicts the shift of the absorption edge for LN:Zr,Mg crystals as a function of MgO doping concentration. Here, the absorption edge is defined as the wavelength where the absorption coefficient is equal to 20 cm^−1^ [27]. Notably, the absorption edge has a violet-shift below the threshold but a red-shift above the threshold with increasing doping [28]. We can see the aforementioned characteristics at the threshold from this figure; in other words, the total doping concentration of 1.5 mol % ZrO_2_ and 3.0 mol % MgO in LiNbO_3_ has reached the threshold level, which agrees with the results of the OH^−^ absorption spectra. In addition to this, the absorption edge of LN:Zr,Mg_3.0_ is far from that of LN:Zr_2.0_, implying that MgO co-doping may induce greater lattice distortion in crystals.

The ion-polarization model can be used to explain the shift of the absorption edge of doped LN [29,30]. In this model, the polarization ability of doped ions is reflected by (Z*)^2^/r, where Z* is the valid nuclear charge number, and *r* is the ionic radius. The intensity of the Nb-O band—that is, the change of the distribution of the O^2−^ ions electron cloud—directly affects the position of the absorption edge. When the doped ions make the polarization of O^2–^ increase, the energy of the electron transition from O^2−^ 2*p*-state to Nb^5+^ 4*d*-state decreases, causing the indirect transition absorption edge to move towards the infrared. Conversely, when the doped ions make the polarization of O^2−^ decrease, the energy of the electron transition from O^2−^ 2*p*-state to Nb^5+^ 4*d*-state increases, causing the indirect transition absorption edge to move towards the ultraviolet. The calculated values of the polarization ability of Li^+^, Nb^5+^, Mg^2+^ and Zr^4+^ are 7.6, 260.0, 78.1, and 196.7, respectively. Special mention should be made that Zr^4+^ and Mg^2+^ have the same ionic radius (0.72 Å), and the electronegativity of Zr^4+^ (1.3) and Mg^2+^ (1.2) is less than that of Nb^5+^ ions (1.6). Based on the Li-vacancy model [31], the doped ions in LN are assumed preferably to replace ${\mathrm{Nb}}_{\mathrm{Li}}^{4+}$ defects until all ${\mathrm{Nb}}_{\mathrm{Li}}^{4+}$ are substituted, and above the doping threshold value, the doped ions occupy the normal Nb and Li sites. Moreover, it is reported that for LN:Mg with other impurities, co-doping Mg^2+^ ions are assumed to affect the site occupancy by removing ${\mathrm{Nb}}_{\mathrm{Li}}^{4+}$ and the impurity ions occupying Li sites [32]. Thus, the doping mechanism for the investigated LN:Zr,Mg is that Zr^4+^ ions replace ${\mathrm{Nb}}_{\mathrm{Li}}^{4+}$ ahead of Mg^2+^; when ${\mathrm{Nb}}_{\mathrm{Li}}^{4+}$ defects are totally substituted by Zr^4+^ and Mg^2+^, the surplus Mg^2+^ ions replace ${\mathrm{Zr}}_{\mathrm{Li}}^{3+}$ and the normal Li sites with increased MgO doping concentration; and Mg^2+^ ions begin to occupy Nb sites after all ${\mathrm{Zr}}_{\mathrm{Li}}^{3+}$ are pushed to Nb sites. According to the analyzed mechanism, we hold that Mg^2+^ and Zr^4+^ ions replacing ${\mathrm{Nb}}_{\mathrm{Li}}^{4+}$ enable the absorption edge to move towards the infrared, while Mg^2+^ ions substituting the normal Li sites make the absorption edge move towards the ultraviolet as the MgO doping concentration increases from 3.0 mol % to 6.0 mol %.

In addition, it can be derived from the above analysis that the appearance of ${\mathrm{Mg}}_{\mathrm{Li}}^{+}$ + ${\mathrm{Zr}}_{\mathrm{Nb}}^{-}$ defect pairs is reasonable in the investigated LN:Zr,Mg except LN:Zr,Mg_1.0_, which is definitely in line with the results of OH^−^ absorption spectra. It has been reported that Zr^4+^ ions occupying Nb sites in LN can significantly affect the defect structures and optical properties of LN, which is completely different from other optical-damage-resistant ions occupying Nb sites [7,17]. Therefore, we think that the ${\mathrm{Mg}}_{\mathrm{Li}}^{+}$ + ${\mathrm{Zr}}_{\mathrm{Nb}}^{-}$ neutral complexes play a specific role in the linear tuning of the phase-matching temperature for LN:Zr,Mg; however, more research is needed to find out the specific role of Mg^2+^ and Zr^4+^ ions.

## 4. Conclusions

In summary, we grew a series of LiNbO_3_ crystals co-doping with fixed ZrO_2_ concentration and various MgO concentrations, and investigated their optical properties and defect structures. For LN:Zr,Mg_3.0_, its optical damage resistance at 532 nm was as high as 6.5 × 10^6^ W/cm^2^, while the phase-matching temperature for doubling 1064 nm was as low as 29.3 °C, close to room temperature, which is helpful in achieving an efficient SHG at room temperature by slightly modulating the incident angle of the fundamental beam. In particular, a linear dependence between the phase-matching temperature and the MgO doping concentration was found. According to the linear relationship, we can adjust the phase-matching temperature within a considerable temperature range by the selection of the MgO doping concentration, and it is useful to grow high-quality crystals for room temperature 90° phase matching. Moreover, the spectra analysis elucidates that ${\mathrm{Mg}}_{\mathrm{Li}}^{+}$ + ${\mathrm{Zr}}_{\mathrm{Nb}}^{-}$ neutral pairs may play a specific role in the linear tuning of phase-matching temperature for LN:Zr,Mg. However, further investigation is needed.

## Figures and Tables

**Table 1:** Position of component peaks in the OH^−^ absorption spectra of CLN, LN:Zr_2.0_, and LN:Zr,Mg.

Samples	Position of Peaks (cm^−1^)		
CLN	3468	3481	3490
LN:Zr_2.0_	3475	3485	3495
LN:Zr,Mg_1.0_	3470	3480	3490
LN:Zr,Mg_3.0_	3473	3481	3491
LN:Zr,Mg_4.0_	3474	3482	3493
LN:Zr,Mg_6.0_	3475	3483	3495
